# Reduction-Responsive Chitosan-Based Injectable Hydrogels for Enhanced Anticancer Therapy

**DOI:** 10.3390/ph16060841

**Published:** 2023-06-05

**Authors:** Trung Thang Vu, Sonyabapu Yadav, Obireddy Sreekanth Reddy, Sung-Han Jo, Soo-Bin Joo, Byeong Kook Kim, Eun Ju Park, Sang-Hyug Park, Kwon Taek Lim

**Affiliations:** 1 Department of Smart Green Technology Engineering, Pukyong National University, Busan 48513, Republic of Korea; 2 Department of Display Engineering, Pukyong National University, Busan 48513, Republic of Korea; 3 Department of Biomedical Engineering, Pukyong National University, Busan 48513, Republic of Korea; 4 Institute of Materials Research and Engineering, Agency for Science, Technology and Research, Singapore 138634, Singapore

**Keywords:** click chemistry, disulfide-based cross-linker, chitosan, hydrogels, reduction-responsive

## Abstract

Selective delivery of anticancer drug molecules to the tumor site enhances local drug dosages, which leads to the death of cancer cells while simultaneously minimizing the negative effects of chemotherapy on other tissues, thereby improving the patient’s quality of life. To address this need, we developed reduction-responsive chitosan-based injectable hydrogels via the inverse electron demand Diels–Alder reaction between tetrazine groups of disulfide-based cross-linkers and norbornene groups of chitosan derivatives, which were applied to the controlled delivery of doxorubicin (DOX). The swelling ratio, gelation time (90–500 s), mechanical strength (G’~350–850 Pa), network morphology, and drug-loading efficiency (≥92%) of developed hydrogels were investigated. The in vitro release studies of the DOX-loaded hydrogels were performed at pH 7.4 and 5.0 with and without DTT (10 mM). The biocompatibility of pure hydrogel and the in vitro anticancer activity of DOX-loaded hydrogels were demonstrated via MTT assay on HEK-293 and HT-29 cancer cell lines, respectively.

## 1. Introduction

Recently, cancer has emerged as a major health problem. Chemotherapy is widely used to treat various cancers around the world. Despite this, chemotherapy and anticancer drugs have a high number of side effects, which makes it difficult to treat patients rationally. To combat this issue, it may be helpful to create smart drug delivery systems (DDS) that can keep the drug molecules flowing into tumors while limiting undesirable effects on healthy tissues [1,2,3]. Hydrogels, which have a three-dimensional network structure, have received a lot of attention in a variety of biomedical sectors [4,5]. Many studies have addressed the use of hydrogels in cancer therapy and tissue engineering because of their swelling ability, porous nature, and biocompatibility [6,7,8]. However, conventional hydrogels of fixed shapes typically have constrained dosage forms and applications [9]. Many researchers have been interested in injectable hydrogels because of their potential biomedical applications. These hydrogels were typically formed through a rapid sol–gel phase change or in situ chemical polymerization [10], and they have garnered a lot of attention because of their ability to entrap bioactive agents at specific sites in the body with only a simple syringe injection [11,12]. Furthermore, injectable hydrogels could exhibit instantaneous morphological changes in response to external stimuli, including light, temperature, pH, ionic strength, shear force, electric or magnetic fields, etc., achieving the sustained and controlled release of encapsulated medicines [13,14]. As a result, injectable hydrogels that are also smart would be regarded as a promising candidate of transporting diverse drugs.

The cross-linking of hydrogels can be accomplished either physically or chemically [15]. Hydrogels that undergo physical cross-linking are held together primarily through noncovalent interactions, such as ionic and hydrogen interactions [16]. Hydrogels that are chemically cross-linked are held together primarily by covalent bonds, which are considerably stronger than physical cross-linking. Chemical cross-linking occurs in three main ways: through click chemistry, free-radical polymerization, and enzyme-induced cross-linking [17]. In comparison to hydrogel networks formed through physical cross-linking, those using chemical cross-linking have superior mechanical properties and stability [18]. For almost a century, scientists have tried to develop a universal cross-linking technique. The system has reached a highly advanced stage, making it difficult to find technological advances. “Click” chemistry was only recently discovered, and it is still in the early stages of development. Because of its great selectivity, rapid reaction rate, and high research value, it offers significant benefits for hydrogel cross-linking. As a result, it has garnered a substantial amount of attention for its application in the synthesis of hydrogels and has grown to become one of the synthetic methods that is utilized the most frequently [19]. The Diels–Alder (DA) reaction is the most desirable of all click chemistry reactions because it occurs without the aid of a catalyst or coupling agent, is extremely selective, and does not generate any unwanted byproducts. This reaction is well-established and is most commonly employed in maleimide–furan chemistry [20]. It has many applications, but its most common usage is in the realm of hydrogels, where it is utilized for applications such as bioactive agent delivery, cell encapsulation, and tissue regeneration [21,22,23]. Recently, the inverse electrone demand Diels–Alder (IEDDA) reaction, which has a quicker reaction rate and greater irreversibility compared to the conventional DA reaction, was developed [24].

**Scheme 1:**
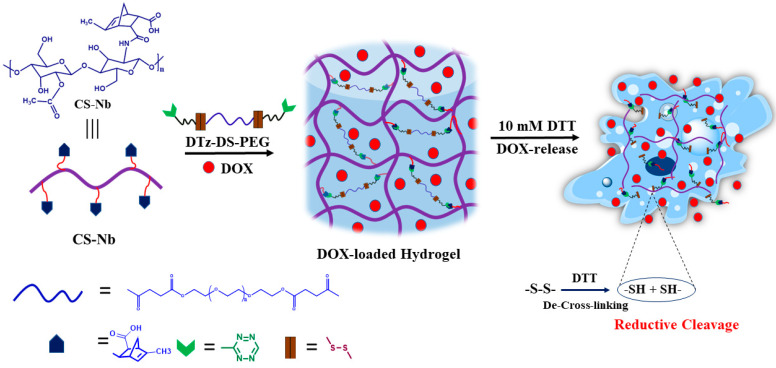
Schematic representation of reduction responsive chitosan–based injectable hydrogels.

Over the years, various types of hydrogels have been generated; however, many of them release drug molecules immediately and at non-targeted sites, which might cause adverse side effects. This issue was exacerbated by the fact that the hydrogel stability was typically weak and unresponsive to tumors. The most crucial factor in the creation of injectable hydrogels for cancer therapy is their ability to respond to the tumor environment [25]. The tumor tissue contains glutathione (GSH, a reducing agent) at a 4-fold higher level than normal tissues [26]; therefore, reduction-responsive hydrogels are of high practical importance for cancer treatment. The objective of this research is to develop novel injectable and reduction-responsive hydrogels by using chitosan (CS) and disulfide cross-linkers. Researchers have made progress in bioactive agent delivery studies by using natural polymers [27,28]. For instance, chitin and its derivatives have been used in the delivery of active pharmaceutical ingredients and dietary supplements [29,30]. CS is one of the derivatives of chitin that has received the most attention from researchers. It possesses a number of advantageous properties, such as antibacterial activity, the capacity to form a film, the ability to interact with a variety of substances, and a high solubility in acidic aqueous media [31,32]. These characteristics make it possible for CS and its derivatives to be utilized in drug delivery applications. In the present work, hydrogels were created by performing the IEDDA reaction, which involved the click reaction between a norbornene (Nb)-substituted CS (CS-Nb) and a water-soluble disulfide cross-linker. The “click” reaction that occurs between Nb and tartrazine (Tz) in water is a crucial factor in the generation of hydrogels with a highly porous and non-toxic nature. A polyethylene glycol (PEG)-type disulfide cross-linker was developed in order to make a precursor that is water-soluble, reduction-responsive, and biocompatible. Scheme 1 shows the schematic route of the creation of the cross-linker and the development of injectable hydrogels. The developed hydrogels exhibited favorable mechanical characteristics and high swelling ratios. The hydrogels had the potential to control the rate at which drugs were released into a tumor’s bloodstream. Results from the cell culture experiment demonstrated that both the cross-linker and the hydrogels were safe for use with fibroblast cells. On the other hand, hydrogels encapsulated with DOX triggered anti-tumor activity in cancer cells. The hydrogels have inherent anticancer properties, are highly efficient at loading agents, and warrant further exploration for use in cancer treatment.

## 2. Results and Discussions

### 2.1. The Preparation of DTz-DS-PEG

**Figure 1:**
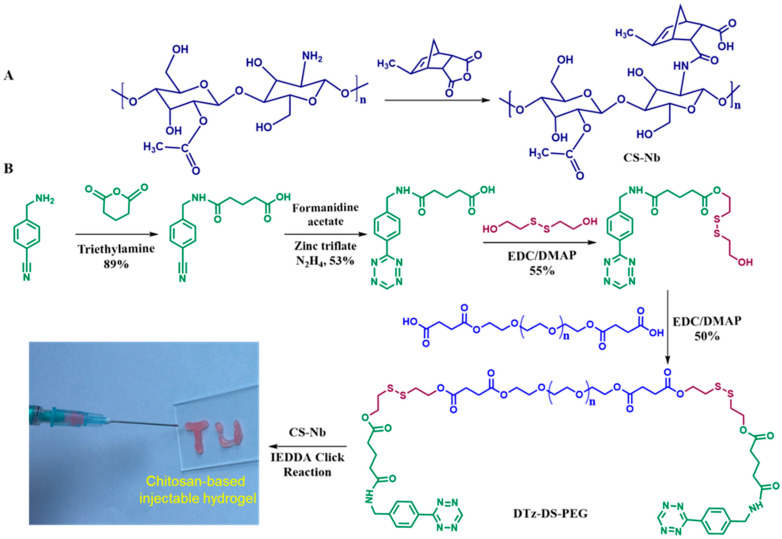
Schematic illustration for the preparation of CS-Nb (**A**), DTz-DS-PEG and reduction responsive chitosan-based injectable hydrogels (**B**).

Figure 1B depicts the synthetic route for the DTz-DS-PEG cross-linkers. In order to produce the cross-linker, an esterification process was executed between PEG-COOH and DS-Tz, with EDC and DMAP as coupling reagents.

**Figure 2:**
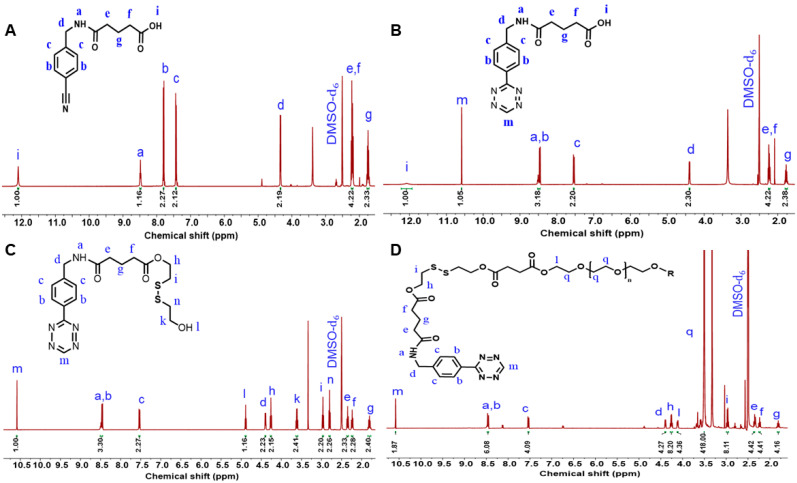
^1^H-NMR spectra of 5-(4-(cyano)benzylamino) 5-oxopentanoic acid (**A**), Tz-COOH (**B**), DS-Tz (**C**), and DTz-DS-PEG (**D**).

^1^H-NMR spectroscopy was used to confirm (4-(cyano) benzylamino)-5-oxopentanoic acid (Figure 2A). The spectrum of (4-(cyano) benzylamino)-5-oxopentanoic acid shows signals at 7.78 and 7.42 ppm, which are attributed to aromatic protons. The signals at 12.05 and 8.44 ppm are attributed to the OH and NH protons of aliphatic chains. The tetrazine group of Tz-COOH is confirmed by a signal at 10.58 ppm in the ^1^H-NMR spectrum (Figure 2B). When comparing the ^1^H-NMR spectra of DS-Tz to that of Tz-COOH, four new signals occur at 4.86, 4.36, 2.93, and 2.76 ppm (Figure 2C). These new signals are attributed to the -CH_2_ protons of disulfide -O-CH_2_-CH_2_-S-S- and -S-S-CH_2_-CH_2_-OH groups, respectively. This proves that the mono-substitution reaction has taken place in the appropriate manner. In the ^1^H-NMR spectra of DTz-DS-PEG (Figure 2D), the proton signals that are characteristic of the disulfide are shifted slightly and combined into a single new signal at 2.97 ppm. The typical proton signal of PEG (-CH_2_-O-CH_2_-) is observed at 3.51 ppm. In addition, the disappearance of the signal corresponding to the -OH of DS-Tz at 4.85 ppm indicates that the esterification reaction and synthesis of DTz-DS-PEG were successful.

### 2.2. Preparation of CS-Nb

**Figure 3:**
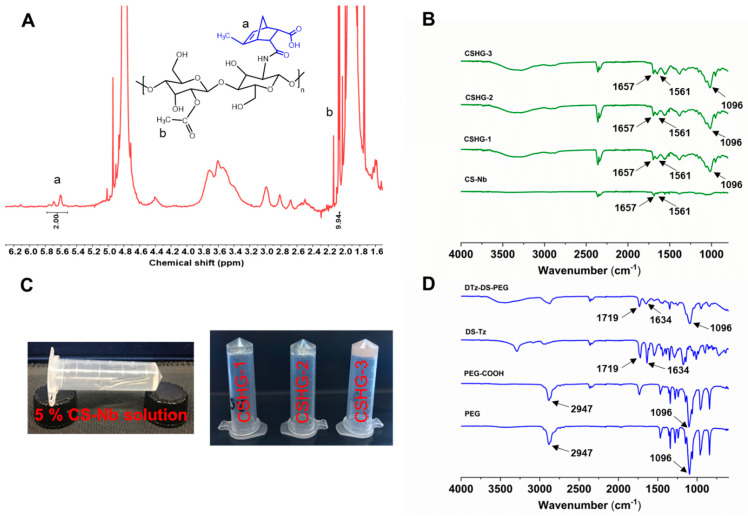
^1^H-NMR spectra of CS-Nb (**A**), ATR-FTIR spectra of CS-Nb, CSHG-1, CSHG-2, and CSHG-3 (**B**), Images to show sol–gel states of formulated hydrogel (**C**), ATR-FTIR spectra of the disulfide cross-linkers and precursors (**D**).

The reaction between CS and methyl-5-norbornene-2,3-dicarboxylic anhydride generated the Nb functionalized CS. The ^1^H-NMR spectra of CS-Nb is shown in Figure 3A, and it reveals that the alkene proton signals of Nb groups arise between 5.9 and 6.4 ppm. The degree of substitution was determined to be 32% by integrating the relevant Nb signals (5.9–6.4 ppm) and comparing the integration to the methyl signals (2.1 ppm) of CS. The FTIR spectra of PEG show the characteristic peak at 1096 cm^−1^ which is assigned to the -CH_2_-O-CH_2_- stretching frequency (Figure 3D). The FTIR spectra of DS-Tz show a peak at 1634 and at 1719 cm^−1^ which correspond to the amide C=N stretching frequency of the Tz group and the C=O stretching frequency of the ester group (Figure 3D). In the case of DTz-DS-PEG, peaks similar to those of DS-Tz are observed. In addition, new peaks at 1096 and 3300–3400 cm^−1^ are found, which correspond to the -CH_2_-O-CH_2_- and -O-H stretching frequencies of the PEG-COOH. This indicates that a successful esterification reaction occurred between PEG-COOH and DS-Tz. The preparation of CS-Nb and hydrogels was further confirmed via FTIR analysis (Figure 3B). The FTIR spectra of CS-Nb show the peaks at 1561 and 1675 cm^−1^ which are assigned to N-H bending and C=O stretching frequencies of amine and amide I groups. The hydrogel spectra also show similar peaks to those of CS-Nb. Along with these peaks, a new peak at 1096 cm^−1^ is found, which is a typical peak of PEG. This confirms the interaction between the Tz group of the cross-linker and the Nb group of the polymeric matrix to form hydrogels.

### 2.3. Characterization of Hydrogels

**Figure 4:**
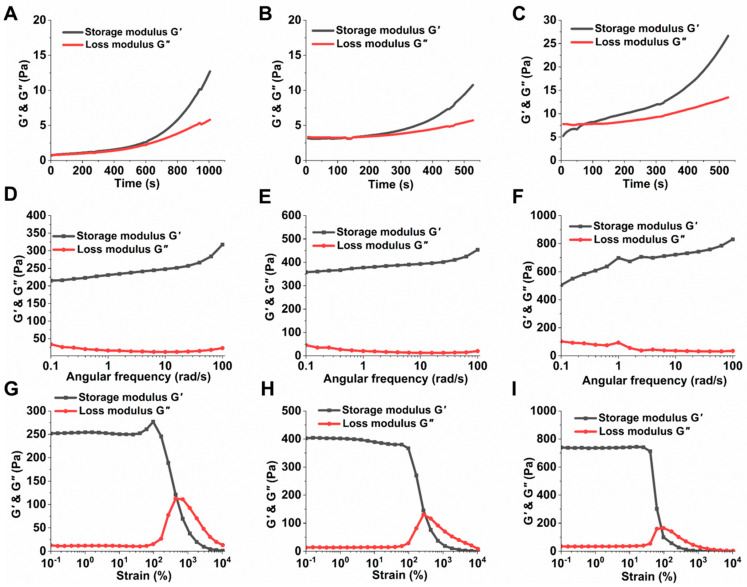
Characterization of the CSHG-1, CSHG-2, and CSHG-3. The loss and storage moduli, function of time, (**A**–**C**); frequency sweep (**D**–**F**); strain sweep (**G**–**I**).

The hydrogels CSHG-1, CSHG-2, and CSHG-3 were formed via the IEDDA reaction between CS-Nb and DTz-DS-PEG (Table 1). The invert-vial test was used to record the times that hydrogels underwent their sol–gel transformation. Figure 3C displays the images that illustrate the sol–gel transformation. When using injectable hydrogels as a drug delivery carrier, the gelation period is essential. We utilized a hybrid rheometer and evaluated the mechanical characteristics of the hydrogels to obtain an exact reading on the amount of time required for the gelation of each hydrogel. Figure 4A–C illustrate that the hydrogels CSHG-1, CSHG-2, and CS-HG-3 formed after 500, 200, and 90 s, respectively. With these gelation times, the pre-gel solution can be mixed and injected into the body, and then the solid gel can form, confirming that the created hydrogels are injectable [33].

The storage modulus (G’) of the hydrogel CSHG-3 achieves a maximum value of 850 Pa, which corresponds to a high degree of cross-linking. Conversely, the storage modulus of the hydrogel CSHG-1 is the lowest (350 Pa). This finding suggests that a higher cross-linker-to-hydrogel ratio increases the storage modulus. As seen in Figure 4G–I, the mechanical characteristics of all the hydrogels remain stable when the strain is less than 10%, proving that the hydrogels have excellent mechanical qualities. The storage modulus of hydrogels began to decrease progressively as the strain increased from 100% to 1000%.

### 2.4. Swelling Properties

**Figure 5:**
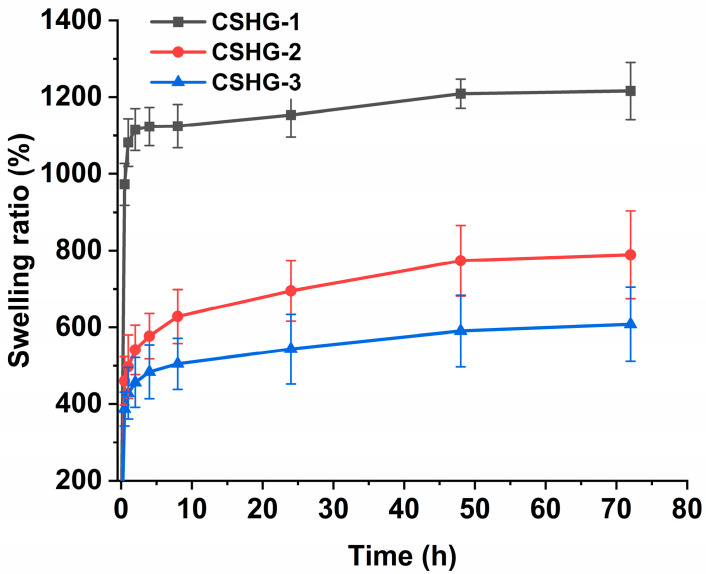
Swelling ratio of CSHG-1, CSHG-2, and CSHG-3 in PBS pH 7.4.

The quick swelling of the hydrogels during the initial phase is most likely caused by the PEG chains of the cross-linker becoming hydrated. The findings corroborate other studies [34,35], where PEG chains could greatly alter the swelling of hydrogels. Furthermore, from Figure 5, it was observed that the hydrogel CSHG-1 showed a higher degree of swelling (1200%) than the hydrogels CSHG-2 and CSHG-3. The lesser swelling of the hydrogels CSHG-2 and CSHG-3 can be attributed to a higher quantity of cross-linkages being present in these hydrogels. This higher amount of cross-linker causes more compact networks inside the hydrogels, which prevents water from permeating the hydrogels [36]. The time and rate at which hydrogels swell are essential factors that can have a significant effect on drug-loading and drug release kinetics. Rapid hydrogel formation allows for greater drug capture within the polymeric matrix. Hydrogels with a high swelling degree can increase the release rate because, if the degree of swelling is high, the hydrogel matrix allows water molecules to diffuse more easily into the hydrogel networks.

**Figure 6:**
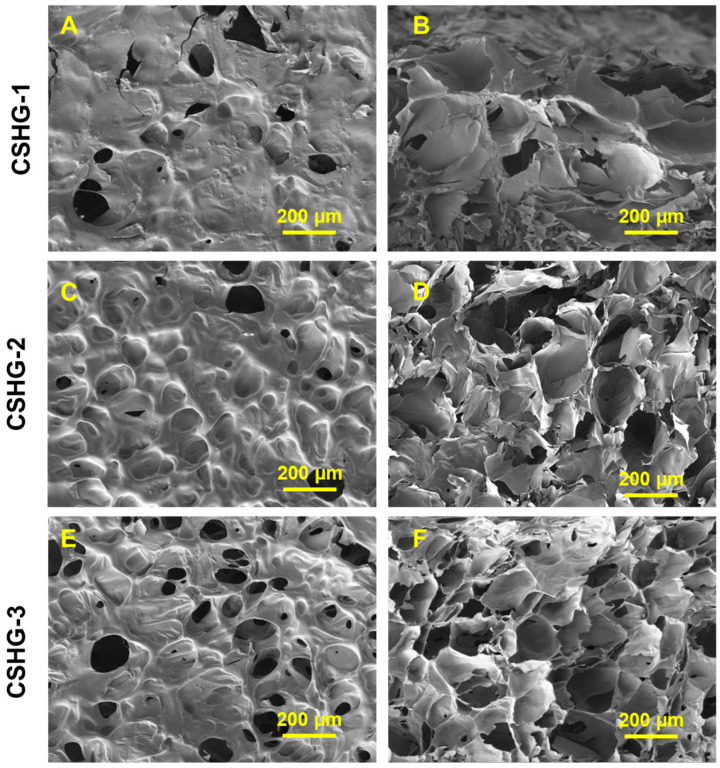
Surface and cross-section SEM images of the hydrogel CSHG-1, CSHG-2, and CSHG-3, shown in (**A**,**C**,**E**) and (**B**,**D**,**F**), respectively.

SEM analysis was used to examine the surface and cross-sectional area of the hydrogel, and the findings are shown in Figure 6. From the SEM images, it is observed that the hydrogel have a micrometer-scale porous nature. The size of pores in CSHG-3 is smaller but the number of pores is higher than those of CSHG-2 and CSHG-1. The high porosity may stem from trapped nitrogen gas (N_2_) generated during the IEDDA reaction between Tz and Nb groups. The porous structure revealed that N_2_ gas might play a significant role in the in situ creation of pores. The porosity of hydrogels is one of their most notable physical features and an essential factor in drug delivery systems. The interconnected porous structures within the hydrogel matrix provide a huge internal area, which can increase water absorption into the networks via capillary force. As a result, the hydrogels might expand quickly and perhaps improve drug delivery.

### 2.5. Studies on Drug-Loading and Release

Table 1 summarizes the drug-loading efficiencies of the hydrogels CSHG-2 and CSHG-3, which are shown to be 92.66 ± 0.64% and 94.31 ± 0.45%, respectively. It was found that the drug-loading efficiency was improved by elevating the cross-linker concentration, probably due to the compact network structure of the resulting hydrogels. Additionally, the higher porosity could contribute to the increased drug loading [37,38]. The higher drug-loading capacity of the hydrogels could be advantageous for the treatment of various types of cancer.

**Figure 7:**
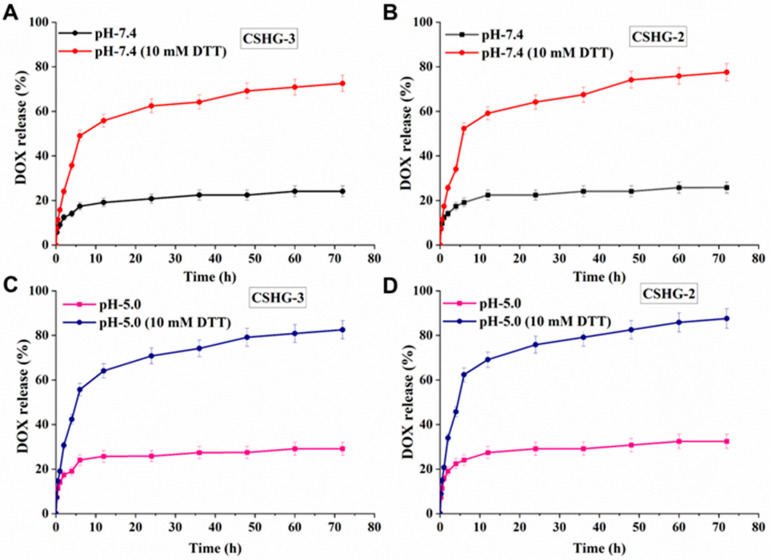
Accumulative DOX release of CSHG-3 and CSHG-2 in PBS pH 7.4 with and without DTT (**A**,**B**) and in PBS pH 5.0 with and without DTT (**C**,**D**), respectively.

The drug release studies of hydrogels were performed under different pHs (7.4 and 5.0). As shown in Figure 7B, the release percentage of DOX from CSHG-2 was about 25% at pH 7.4 after 72 h in the absence of DTT. This result demonstrates that hydrogels are stable and have minimal pre-release of DOX at the physiological pH of 7.4. On the other hand, the disulfide bond in the cross-linkages of hydrogels was broken by DTT via a reductive process, which caused the DOX molecules to leak out quickly, increasing the percentage of DOX released from CSHG-2 to 52% for the first 6 h and reaching 77% over the same time period. Consequently, the DOX molecules leaked out quickly [39]. The release rate of DOX from CSHG-3 at pH 7.4 (with and without DTT, Figure 7A) was slightly lower than that of the CSHG-2 hydrogel. This is due to the fact that the increased cross-linking led to smaller mesh sizes, which in turn produced hydrogels with more compact structures; consequently, the release rate decreased [40]. At an acidic pH of 5.0 (Figure 7D), the release behavior of DOX from CSHG-2 was 32% after 72 h. In the presence of DTT, the release behavior of DOX from CSHG-2 increased by 62% for the first 6 h and reached 88% at 72 h. The release rate of DOX from CSHG-3 at pH 5.0 (with and without DTT, Figure 7C) was slightly lower than that of the CSHG-2 hydrogel. From these results, it was clearly understood that a lower release rate was observed in the hydrogels at both pHs (7.4 and 5.0) without DTT. Since the percentage of DOX released in 10 mM DTT at acidic pH 5.0 (88%) was higher than at neutral pH 7.4 (77%), DOX released preferentially in the acidic condition, probably owing to the protonation of DOX [41]. Hence, the developed hydrogels showed reduction responsive and controlled release behavior. This type of reductively responsive hydrogel has potential for anticancer treatment and drug delivery applications.

### 2.6. Cytotoxicity Assay

**Figure 8:**
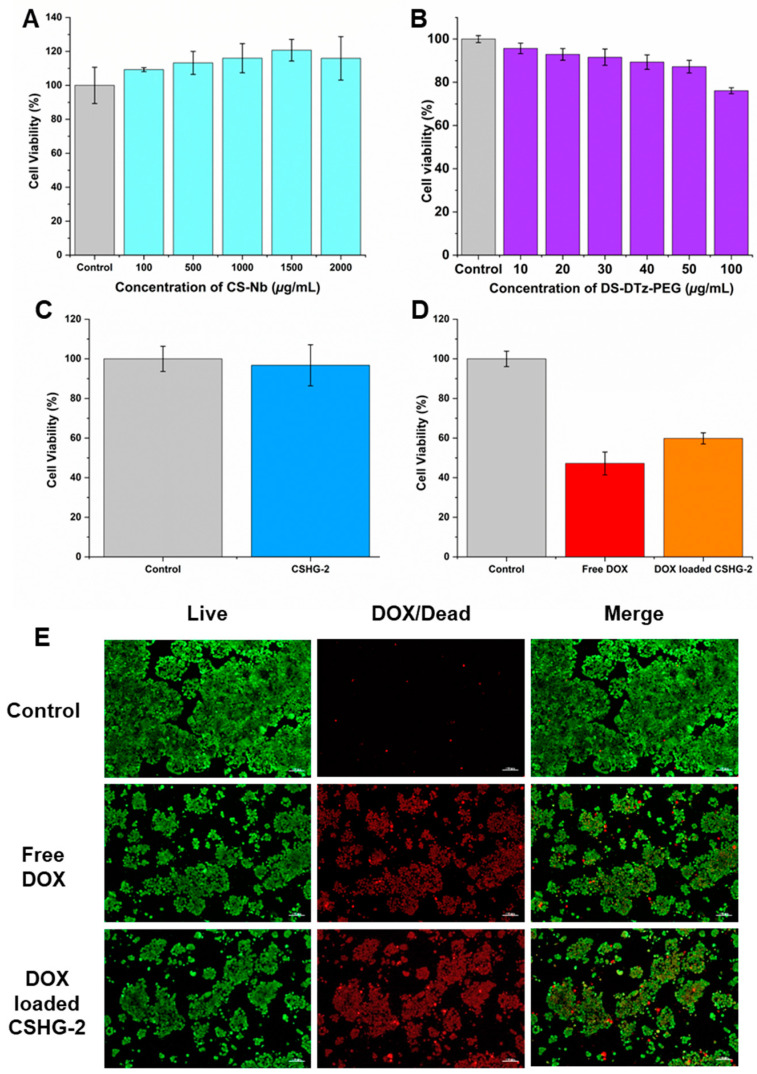
Cytocompatibility of CS-Nb (**A**), DS-DTz-PEG (**B**), empty hydrogel examined in HEK-293 cells (**C**), in vitro anti-tumor activity in HT-29 cells following treatment with DOX-loaded CSHG-2 and free DOX (10 µg) (**D**), and microscopic images showing antitumor effect of DOX-loaded CSHG-2 and free DOX (live cells and dead cells were represented by green color and red color, respectively) (**E**). Scale bars showing 100 µm.

The MTT assay was used to evaluate the cytotoxicity of CS-Nb, DTz-DS-PEG, and pure hydrogels, and the results are depicted in Figure 8A–C. Hydrogels, the DTz-DS-PEG cross-linker, and CS-Nb had negligible effects on HEK-293 cell proliferation. In particular, HEK-293 cell viabilities treated with varying concentrations of CS-Nb and DTz-DS-PEG were greater than 70% at all the concentrations tested. These findings demonstrate that CS-Nb, CSHG-2, and the DTz-DS-PEG cross-linker are all safe for use around healthy cells. The DOX-loaded CSHG-2 were tested for them in vitro antitumor activity against HT-29 cancer cells, and the results were compared to those obtained with free DOX (Figure 8D). After 24 h of treatment, the DOX-loaded CSHG-2 induced apparent cytotoxicity in HT-29 cancer cells. It is also notable that the DOX-loaded hydrogels had slightly less potent cytotoxic effects on HT-29 cells than free DOX. This is because the drug in the hydrogel is released gradually over time. This result is consistent with the findings of previous studies [42,43], which discovered that the toxicity of a drug decreased when it was released slowly over a period of time. The effect of hydrogels on HT-29 cancer cells was further investigated by live/dead images (Figure 8E). As compared with the control, free DOX and DOX-loaded CSHG-2 caused cytotoxicity in HT-29 cells (as shown by red-dots). The above results indicate that hydrogels could be a good candidate for controlled drug delivery applications.

## 3. Materials and Methods

### 3.1. Materials

Succinic anhydride (98%), 1-ethyl-3-(3-dimethylaminopropyl)-carbodiimide hydrochloride (EDC.HCl, 99%), glutaric anhydride (98%), 4-(aminomethyl)benzonitrile hydrochloride (98%), CS (Mw = 80 kDa, 70% deacetylation degree), formamidine acetate (98%), and zinc (II) trifluoromethanesulfonate (99%) were purchased from Tokyo Chemical Industry. Bis(2-hydroxylethyl) disulfide (90%, Alfa Aesar). Sodium nitrite (97%), PEG 4000, methyl-5-norbornene-2,3-dicarboxylic anhydride (99%) triethylamine (TEA, 99%), 4-(dimethylamino)pyridine (DMAP, 99%), 1,4-dithiothreitol (DTT, 98%), N-hydroxysuccinimide (NHS, 98%), and silica gel powder (230–400 mesh) were bought from Sigma Aldrich. DMSO, hexane, acetonitrile, dichloromethane (DCM), ethyl acetate, and diethyl ether were obtained from SK Chemicals. Polyethylene glycol carboxylic acid (PEG-COOH) was prepared by the method in our previous paper [33].

### 3.2. Preparation of Reduction-Responsive Cross-Linkers, DTz-DS-PEG

The preparation of DS-Tz was described in the supplementary information. PEG-COOH and DS-Tz facilitated the synthesis of the cross-linker (DTz-DS-PEG). A typical process involves dissolving PEG-COOH (0.378 g, 0.09 mmol) in DCM (500 mL) in a round bottom flask, then cooling the mixture to 0 °C in an ice bath to prevent any unintended reactions. The reaction was conducted in a nitrogen atmosphere. To the reaction mixture (RM), EDC.HCl (0.087 g, 0.45 mmol) and DMAP (0.022 g, 0.18 mmol) were added. After 1 h of stirring, 3 mL of DS-Tz (0.12 g, 0.228 mmol) in DCM was slowly added to the RM, and then the ice bath was withdrawn. The RM was stirred at room temperature for three days while being monitored with TLC. Afterwards, the solution was rinsed three times with 50 mL of deionization (DI) water and 50 mL of brine. The organic phase DCM was collected and concentrated under a vacuum condition. The final step involved using a methanol-in-DCM (7%) column chromatography system to isolate the product. A 50% yield was discovered. ^1^H-NMR (400 MHz, DMSO-*d_6_* ppm): *δ* = 1.80 (dd, 4 H), 2.24 (t, 4 H), 2.35 (t, 4 H), 2.57 (s, 8 H), 2.97 (dt, 8 H), 3.51 (s, 182 H), 4.26 (q, 8 H), 4.12 (t, 4 H), 4.40 (d, 4 H), 4.40 (d, 4 H), 7.53 (d, 4 H), 8.48–8.44 (m, 6 H), 8.48–8.44 (m, 6 H), 10.58 (s, 2 H).

### 3.3. Preparation of CS-Nb

A quantity of 0.5 g of CS was mixed in 50 mL of a 2% acetic acid solution, and the mixture was stirred for 3 h at room temperature to achieve homogeneity. The mixture was then cooled to 0 °C. Afterward, 0.47 mL of methyl-5-norbornene-2,3-dicarboxylic anhydride in 3 mL of DMSO was added dropwise and the solution was stirred at 60 °C for a day. 1 M NaOH solution was added to bring the pH of the mixture to 7. Next, the mixture was precipitated in 250 mL of acetone to collect a solid. The solid product was resuspended in 50 mL of 2% acetic acid solution and dialyzed again in DI water using a dialysis membrane (MWCO = 12–14 KDa) for 2 d. Finally, the solution was lyophilized to obtain CS-Nb.

### 3.4. Preparation of Hydrogels

A quantity of 100 µL of CS-Nb (5% *w*/*v*) in PBS was placed in a vial and stirred until it became homogenous. Meanwhile, 30 µL of PBS was used to dissolve at various concentrations of the DTz-DS-PEG cross-linker based on the Nb/Tz ratio (4/1, 2/1, and 1/1, respectively; these concentrations were labelled (CSHG-1, CSHG-2, and CSHG-3). The DTz-DS-PEG solution and the CS-Nb solution were combined in a vortex mixer to generate hydrogels.

### 3.5. Drug Loading

The following steps were performed to load the drug into the hydrogels. A solution of CS-Nb was initially mixed with a solution containing 1 mg/mL DOX in PBS. Then, DTz-DS-PEG was added to the DOX/CS-Nb combination, and it was stirred using a vortex mixer for 10 s. After the hydrogels were freeze-dried, any unloaded DOX was extracted by soaking the dry hydrogels in PBS. The supernatant was assessed using UV-vis spectrophotometry at a wavelength of 485 nm to ascertain the efficacy of drug loading. The formula below was used to calculate the drug loading efficiency percentage:

(1) $$\mathrm{Loading}\;\mathrm{efficiency}\;(\%)=\frac{(\mathrm{quantity}\;\mathrm{of}\;\mathrm{DOX}\;\mathrm{in}\;\mathrm{feed}\text{-}\mathrm{quantity}\;\mathrm{of}\;\mathrm{DOX}\;\mathrm{in}\;\mathrm{supernatant})}{\left(\mathrm{quantity}\;\mathrm{of}\;\mathrm{DOX}\;\mathrm{in}\;\mathrm{feed}\right)}\;\times 100$$

### 3.6. Swelling Property

A gravimetric approach was used to determine the swelling ratios. The dry hydrogels were immersed in PBS with a pH of 7.4. After a predetermined amount of time, tissue paper was used to carefully remove any extra water that had accumulated on the hydrogel surface. We used an analytical balance to measure the hydrogel masses. The swelling ratio was calculated using the following formula:

(2) $$\mathrm{SR}\;(\%)=\frac{M_{s}-M_{d}}{M_{d}}\;\times 100$$

where M_d_ and M_S_ are the mass of dry and swollen hydrogels, respectively, at various points in time.

### 3.7. Drug Release

First, unloaded DOX was removed by washing freeze-dried hydrogels in PBS. The hydrogels were placed in a dialysis bag (MWCO of 3500) in PBS containing 5 mL of 10 mM of DTT or PBS (pH 7.4 and pH 5.0) and were dialyzed in 30 mL of a respective medium at 100 rpm and 37 °C. A UV-vis spectrophotometer with a 485 nm wavelength was used to analyze 3 mL of samples from each group at predefined intervals. The buffer was replenished with 3 mL of buffer medium after each test to keep the volume constant. The percentage of drug release was calculated using the following equation.

(3) $$\mathrm{Drug}\;\mathrm{release}\;(\%)=\frac{Total\;released\;DOX}{Total\;DOX\;in\;hyrogels}\;\times 100$$

## 4. Conclusions

In the present work, the disulfide cross-linker DTz-DS-PEG was used to develop CS-based injectable hydrogels. The IEDDA reaction rapidly took place at room temperature to chemically cross-link CS-Nb with DS-DTz-PEG. The Tz-Nb click reaction resulted in the production of N_2_ gas, which acted as a porosity-creating agent in the hydrogel matrix. The drug-loading capability of the porous hydrogels was found to be exceptionally high (≥92%). In addition, the PEG chain that is a part of the DTz-DS-PEG cross-linker not only made it possible for the cross-linker to be soluble in water but was also a primary factor in the quick and significant swelling (1200% in pH 7.4). Compared to the PBS solution without DTT, the hydrogels released significantly more DOX (~77%) in the 10 mM DTT solution (after 72 h at pH 7.4), showing typical reduction responsiveness. The DOX release was further enhanced in the acidic condition (~88% at pH 5). Pure hydrogels exhibited no discernible impact on HEK-293 fibroblast cells, but the DOX-loaded hydrogels caused distinct cytotoxicity in HT-29 cancer cells. The injectable hydrogels made of chitosan may make attractive candidates for controlled drug delivery applications involving stimuli-responsive drug release.

## Figures and Tables

**Table 1:** Hydrogel formulation, gelation time, and drug-loading efficiency.

Samples	Ratio Nb/Tz	Gelation Time (s)	Drug Loading Efficiency (%)
CSHG-1	4/1	500	Not applicable
CSHG-2	2/1	200	92.66 ± 0.64
CSHG-3	1/1	90	94.31 ± 0.45

## Data Availability

Data is contained within the article and supplementary material.

## Supplementary Materials

The following supporting information can be downloaded at: https://www.mdpi.com/article/10.3390/ph16060841/s1. Preparation and characterization techniques in supplementary information.
