# Supplementary material for: Reduction-Responsive Chitosan-Based Injectable Hydrogels for Enhanced Anticancer Therapy
Source: Pharmaceuticals (Basel). 2023 Jun 5;16(6):841. doi: 10.3390/ph16060841 (PMC10304555; doi:10.3390/ph16060841)
Supplement: Supplementary file 1 [file pharmaceuticals-16-00841-s001.zip › pharmaceuticals-2425184-supplementary.pdf]

Supplementary Information

Reduction-responsive chitosan-based injectable hydrogels for enhanced anticancer therapy

Trung Thang Vu[1 ‡], Sonyabapu Yadav[1 ‡], Obireddy Sreekanth Reddy[2], Sung-Han Jo[3], Soo-Bin Jo[1], Byeong Kook Kim[3], Eun Ju Park[4], Sang-Hyug Park[3], Kwon Taek Lim[1, 2,*]

[1] Department of Smart Green Technology Engineering, Pukyong National University, Busan, 48513, South Korea

[2] Department of Display Engineering, Pukyong National University, Busan, 48513, South Korea

[3] Department of Biomedical Engineering, Pukyong National University, Busan, 48513, South Korea

[4] Institute of Materials Research and Engineering, Agency for Science, Technology and Research, Singapore 138634

* Corresponding author: K. T. Lim (E-mail: ktlim@pknu.ac.kr)

‡ These authors contributed equally

Characterization

A TESCAN (MIRA 3 LMH) Field Emission scanning electron microscope (FE-SEM) was used to observe the morphologies of hydrogels. ^{1}H-NMR spectra were investigated using a Nuclear Magnetic Resonance instrument (JNM ECZ-400, JEOL). A UV–vis spectrophotometer (Optizen POP, Optizen) was used to record UV-vis spectra with the measurement range of 400-600 cm^{-1} FTIR spectra were obtained using a Fourier transform infrared spectrometer (CARY 640, Agilent). The mechanical properties of hydrogels were studied using a hybrid rheometer (DHR-2, TA instrument). The viscous loss modulus and elastic storage modulus of hydrogels were determined at continuous strain of 5%, frequency sweep from 0.1 to 100 rad/s, and strain sweep from 0.1% to 10000%.

Synthetic procedure of 5-(4-(1,2,4,5-tetrazin-3-yl) benzylamino)-5-oxopentanoic acid (Tz-COOH).

3 grams of 4-(aminomethyl) benzonitrile hydrochloride was dissolved in 150 mL of acetonitrile and transferred to a dry 2-neck round bottom flask. The flask was purged with nitrogen gas for 30 minutes to remove any moisture and oxygen. Next, 7.5 mL of triethylamine was added to the flask using a glass syringe as a base, and the mixture was stirred for an additional 30 minutes. After that, 2.23 grams of glutaric anhydride, which was dissolved in acetonitrile using a glass syringe, were added to the reaction mixture. The reaction mixture was then heated at 85 ºC for 24 hours. After cooling, the solvent was removed using rotary evaporation, and the resulting solid was dissolved in 150 mL of deionized water. The pH of the solution was adjusted to 3 with 1N HCl, and the mixture was extracted three times with 150 mL of ethyl acetate. The combined organic layers were washed three times with 150 mL of deionized water and 150 mL of brine and then dried over anhydrous magnesium sulfate. The yield of the reaction was 89%, and a white solid was obtained as the final product, which was (4-(cyano)benzylamino)-5-oxopentanoic acid. (^{1}H NMR, 400 MHz, DMSO-d_6, ppm): δ = 1.79–1.69 (m, 2H), 2.26–2.15 (m, 4H), 4.33 (d, J = 6.0 Hz, 2H), 7.42 (d, J = 8.6 Hz, 2H), 7.78 (d, J = 8.5 Hz, 2H), 8.44 (t, J = 5.9 Hz, 1H) 12.05 (s, 1H).

In a dry round bottom flask, a mixture of 2 g of (4-(cyano)benzylamino)-5-oxopentanoic acid, 4.24 g of formamidine acetate salt, and 0.148 g of zinc triflate was prepared. This was followed by the slow addition of 5 milliliters of freshly distilled anhydrous hydrazine, and the mixture was stirred at room temperature for 24 hours to allow for the formation of the desired intermediate. Next, a solution of 5.64 grams of sodium nitrite was added to the flask, and the reaction was cooled to 0 ºC. A 1 N solution of hydrochloric acid was then added slowly until the gas evolution ceased. This step allowed for the conversion of the intermediate to the final product. The mixture was then extracted with dichloromethane, washed with brine, and dried with anhydrous magnesium sulfate to remove any remaining impurities. The final product was obtained by filtering the mixture,

removing the dichloromethane using rotary evaporation, and recrystallizing the compound in cold isopropyl alcohol to improve its purity. The yield of the product was found to be 53%, and it exhibited a distinctive bright pink color. (^{1}H NMR, 400 MHz, DMSO-d_6, ppm): δ = 1.77 (p, J = 7.3 Hz, 2H), 2.28–2.17 (m, 4H), 4.40 (d, J = 5.9 Hz, 2H), 7.53 (d, J = 8.6 Hz, 2H), 8.52–8.40 (m, 3H), 10.58 (s, 1H), 12.06 (s, 1H).

Synthesis of 2-((2-hydroxyethyl)disulfanyl)ethyl 5-((4-(1,2,4,5-tetrazin-3-yl)benzyl)amino)-5-oxopentanoate (DS-Tz)

The mono-substitution reaction was carried out between Tz-COOH and diS-OH, as follows.

Tz-COOH (0.8 g, 2.66 mmol) was dissolved in a mixture of DCM (200 mL) and DMSO (20 mL) in a 2-neck RBF, purged with nitrogen in an ice bath. DMAP (0.46 g, 3.18 mmol) and EDC.HCl (0.612 g, 3.18 mmol) in DCM were introduced into the flask, then it was stirred for 1 h. Afterwards, diS-OH (0.41 g, 2.66 mmol) dissolved in 4 mL of DCM was introduced into the mixture by using a glass syringe at 0 °C. Subsequently, the flask was kept at RT for 3 d under inert atmosphere. Then, the solution was extracted with 3 × 150 mL of deionized (DI) water and washed with brine. The DCM layer was collected after extraction. Afterwards, anhydrous $MgSO_4$ was used to dry the DCM solution, then the solution was filtered, and evaporated to produce a crude mixture. Finally, the product was obtained by column chromatography (5% hexane in ethyl acetate). The total yield from column chromatography was 55%. In ^{1}H-NMR (400 MHz, DMSO-d_6, ppm): δ = 1.80 – 1.73 (m, 2 H), 2.20 (dd, 2 H), 2.31 (t, 2 H), 2.76 (t, 2 H), 2.93 (dd, 2 H), 3.60 – 3.54 (m, 2 H), 4.22 (t, 2 H), 4.36 (d, 2 H), 4.86 – 4.83 (m, 1 H), 7.51 – 7.48 (m, 2 H), 8.47 – 8.40 (m, 3H), 10.54 (s, 1 H).

Cell cytotoxicity assay

The cytotoxicity of CS-Nb, DTz-DS-PEG, CSHG-2, and DOX-loaded CSHG-2 was evaluated using a water-soluble tetrazolium salt (WST) assay. Although the MTT assay is commonly used

for this purpose, it requires dimethyl sulfoxide (DMSO) as a solvent, which we sought to avoid. Instead, we employed a WST assay that is soluble in water. To perform the assay, CS-Nb and DTz-DS-PEG were directly dissolved in Dulbecco's Modified Eagle Medium (DMEM) at varying concentrations and incubated with fibroblast cells for 24 hours. CSHG-2 and DOX-loaded CSHG-2 (0.05 mg/mL) were immersed in DMEM containing 10 % fetal bovine serum and incubated for 24 hours. The resulting extracts from each sample were collected and filtered using a 0.22 μm filter. The extracts were then incubated for 24 hours with HEK-293 fibroblast cells in a 48-well plate with 10^4 cells per well. In a parallel experiment, DOX-loaded CSHG-2 were treated with HT-29 cancer cells in a similar manner as described above. The viability of the cells was assessed using WST assays.

Statistical results

All experiments were performed in triplicate and the data are given as mean ± standard deviation.
